# Producing Soft Magnetic Composites by Spark Plasma Sintering of Pseudo Core–Shell Ni–Fe Alloy@Mn_0.5_Zn_0.5_Fe_2_O_4_ Powders

**DOI:** 10.3390/ma16020501

**Published:** 2023-01-04

**Authors:** Loredana Cotojman, Traian Florin Marinca, Florin Popa, Bogdan Viorel Neamțu, Virgiliu Călin Prică, Ionel Chicinaș

**Affiliations:** Materials Science and Engineering Department, Technical University of Cluj-Napoca, 400641 Cluj-Napoca, Romania

**Keywords:** pseudo core–shell particles, soft magnetic composites, spark plasma sintering

## Abstract

Soft magnetic composite (SMC) cores have been obtained by Spark Plasma Sintering (SPS) using pseudo core–shell powders. Pseudo core–shell powders are formed by a core of soft magnetic particle (nanocrystalline permalloy or supermalloy) surrounded by a thin layer (shell) of nanosized soft ferrite (Mn_0.5_Zn_0.5_Fe_2_O_4_). Three compositions of pseudo core–shell powders were prepared, with 1, 2 and 3 wt.% of manganese–zinc mixt ferrite. The pseudo core–shell powders were compacted by SPS at temperatures between 500 and 700 °C, with a holding time ranging from 0 to 10 min. Several techniques have been used for characterization of the samples, both, powders and compacts X-ray diffraction (XRD, scanning electron microscopy (SEM), energy dispersive X-ray spectroscopy (EDX), magnetic hysteresis measurements (DC and AC) and electrical resistivity. The electrical resistivity is in the order of 1 × 10^−2^ Ωm, 3–4 orders of magnitude higher than supermalloy electrical resistivity. The SPS at lower temperatures (500 °C) conserves the initial phases of the composite, but increasing the sintering temperature and/or sintering time produces a solid-state reaction between the alloy and ferrite phases, with negative consequence on the magnetic properties of the compacts. The initial relative permeability is around 40 and remains constant until to 2000 Hz. The power losses are lower than 2 W/kg until to 2000 Hz.

## 1. Introduction

The soft magnetic composite (SMC) materials are from decades of great interest from both research and applicative point of views. This great group of magnetic materials are a promising way to obtain better soft magnetic materials that can work in high frequencies (this implies high electrical resistivity) with very good magnetic characteristics, such as induction and permeability. The strengthened way of producing such a composite is to cover the ferromagnetic particles with a thin layer of dielectric material and further, to compact the particles with powder metallurgy techniques [1,2,3,4,5,6,7]. The dielectric material layer can be of various types [8,9,10,11,12,13], and the role of this layer is to insulate the ferromagnetic particles and, in some cases, to bind the composite particles from one to another. Such an approach has limitations, especially related to compact density, the heat treatment that can be applied after densification, and the presence of nonmagnetic phases, etc. To counterattack these limitations better densification techniques are needed for having a better density and to change the nature of the dielectric layer. A handled approach for compaction is to use spark plasma sintering (SPS) [14,15,16,17,18,19]. With this technique, the sintering temperature can be lowered, leading to the possibility of sintering composite particles whose phases can react if they were sintered by the classical sintering route. Using this technique, the density of the sintered composites can reach a high value, close to the theoretical one. This technique’s sintering time is very low, which also helps to avoid the reactions among the composite particles’ phases during the process [14,16,17,18]. To change the dielectric nature can be challenging also. A very good idea is to use soft magnetic ferrite to cover the ferromagnetic particles. The soft magnetic ferrites possess very high electrical resistivity and also have good magnetic characteristics, such as permeability and induction [16,17,20,21,22]. By combining the ferromagnetic particles with a dielectric layer formed by ferrites and the use of SPS for powder densification can be an intriguing approach. The densification by SPS of composite powders of the alloy@ferrite type can be difficult, and the sintering procedure parameters should be chosen very carefully to avoid reactions among composite phases [14,16,17,18]. A convenient way to cover the ferromagnetic particles is to use ferrite nanoparticles [6,14]. Mn–Zn ferrite is one of the most used in industries. Its magnetic and electric properties are suitable for use in SMC as a dielectric [23,24,25].

The paper presents results regarding the synthesis through spark plasma sintering of soft magnetic composites of a Ni-based alloy in a manganese–zinc ferrite (Mn_0.5_Zn_0.5_Fe_2_O_4_) resistive matrix. It is a new and simple suitable route that involves an accessible way of covering a Ni-based ferromagnetic alloy with a thin layer of nanoparticles of manganese–zinc ferrite, and it uses a low-energy densification procedure. The combination of ferromagnetic Ni-based particles with ferrite was used for densification through this route until now, but such a combination of precursors is new and to the best of our knowledge was not used before.

## 2. Materials and Methods

Soft magnetic nanocrystalline permalloy (Ni_3_Fe) and supermalloy (79Ni16Fe5Mo, wt. %) were obtained by mechanical alloying, using elemental powders: Fe (NC 100.24, 99.85% purity, produced by Höganäs AB, Höganäs, Sweden), Ni carbonyl 123 (99.9% purity, Alpha Aesar, Kandel, Germany), and Mo (99.95% purity, Alpha Aesar, Kandel, Germany) produced by chemical reduction. The mechanical alloying was carried out for up to 20 h in a planetary ball mill manufactured by Fristch, model Pulverisette 6 (Idar-Oberstein, Germany). The milling/alloying process was done in a high-purity argon atmosphere using a vial of 500 mL and 14 mm diameter balls. The milling time was up to 20 h, and the ball-to-powder mass ratio was 8:1. After the synthesis and characterization of the as-obtained Ni-based alloys, the powder was covered by a thin layer of manganese zinc iron oxide nanoparticles/nanopowder to obtain pseudo core–shell-like composite particles. This combination was chosen because Ni–Fe alloys are well-known to be very good soft magnetic alloys, and Mn–Zn ferrite is also one of the most-used ferrites in industries. The mixed ferrite nanoparticles have the chemical formula Mn_0.5_Zn_0.5_Fe_2_O_4_ and have been supplied by American Elements, Los Angeles, CA, USA. The nanoparticles are 5–100 nm in diameter. The nanoparticles were dispersed in acetone, and then the Ni-based alloy powder was added. The mixture was continuously homogenized until the acetone evaporation. The amount of nanoparticles were 1, 2, and 3 wt.%. After the acetone evaporation, the as-obtained pseudo core–shell-like particles were dried for 24 h at room temperature. These composite particles are named „pseudo core–shell” particles because the shell is formed by a discontinuous layer of nanosized MnZn ferrite, not a continuous layer like in the case of real core–shell particles. The composite particles were subjected to densification by spark plasma sintering (SPS). The sintering process was performed using home-made spark plasma sintering equipment. The sintering process was performed in an argon atmosphere to avoid the supplementary apport of oxygen to the samples and to protect the punches and die. The spark plasma sintering equipment works at a voltage of 24 V and a current of 3.75 kA. The die and punches were manufactured by graphite. A constant pressure of 30 MPa was maintained during the heating–sintering and cooling cycle. The heating rate was about 5 °C/s. The sintering temperatures were 500, 600, and 700 °C. The sintering time was 0 min for the sintering temperatures of 500 and 700 °C (this means only heating + cooling, without maintaining at the sintering temperature). For a sintering temperature of 600 °C, the sintering time was 0, 5, and 10 min. The cooling of the system down to room temperature was done by the installation of a cooling circuit that uses water. Cylindrical samples were obtained by sintering, and after sintering, the specimens were drilled to make them toroidal. Before drilling, the sintered samples were prepared from a metallographic point of view using silicon carbide sandpaper and an alumina solution. The density of the composite compacts was determined by measuring the mass and the geometrical dimensions of the cylindrical compacts (diameter and height). After the obtention of the toroidal samples, they were wired using copper wire of 0.35 mm (secondary coil) and 0.5 mm (primary coil). The structural investigations were done by X-ray diffraction. An Inel Equinox 3000 (INEL, Artenay, France) with a curved detector using Co radiation was utilized. A 2-theta range of 20–110 degrees was investigated in reflection mode. The calculations of crystallite size (D) were performed with Scherrer’s relation, D = K·λ/(β·cos θ). In Scherrer’s relation, K is the shape factor, a dimensionless constant with 0.9 value; λ is the X-ray wavelength; β is the diffraction line broadening at half of the maximum intensity (FWHM—Full Width at Half Maximum), and θ represents the Bragg angle [26]. The microstructural analyses were done by scanning electron microscopy using a Jeol-JSM 5600 LV microscope (Tokyo, Japan). The microscope is equipped with an energy-dispersive X-ray (EDX) detector for local chemical analysis. The EDX detector is a ULTIMMAX65 model, Oxford Instruments (High Wycombe, UK) which works with Aztec software, Version 4.2. A four-point homemade installation was used for electrical resistivity measurements. A computer-controlled hysteresisgraph (Magnet-Physik Dr. Steingroever GmbH, Remagraph–Remacomp C–705 model, (Cologne, Germany)) was used for the DC and AC characterizations of the sintered compacts. The AC-tested frequency range was 50–10,000 Hz, and the induction level was at 0.01 T.

## 3. Results and Discussions

**Figure 1:**
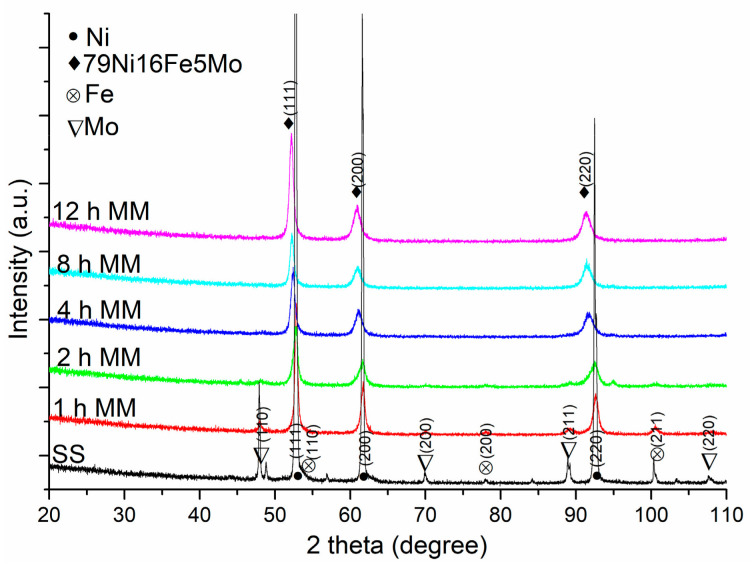
X-ray diffraction patterns of the as-milled samples (1, 2, 4, 8, 12 h) and of the starting sample (ss—0 h milled). For clarity, the XRD patterns have been shifted vertically.

The X-ray diffraction (XRD) patterns of the Ni79Fe16Mo5 (wt. %) samples milled up to 12 h, presented in Figure 1, illustrate progressive supermalloy formation due to the energy transfer from the milling balls to the powders during the milling process. The ss (starting sample), which we named the (Ni–Fe–Mo) starting powders mixture, is given for reference. In Figure 1, the main changes in the XRD patterns are the progressive disappearance of the Fe and Mo Bragg maxima and the displacement of the Ni Bragg maxima to the positions of the supermalloy maxima (lower diffraction angles). From Figure 1 (considering the position and symmetrical shape of the diffraction maxima), it can be seen that, after 8 h of milling, the supermalloy is obtained as a single phase in the milled sample. The same results were obtained for the Ni_3_Fe intermetallic compound. The mean crystallite size of the Ni_3_Fe and supermalloy powders is around 16–18 nm. More information about producing Ni_3_Fe and supermalloy powders by dry or wet mechanical alloying can be found in our previous papers [27,28,29,30].

The nanocrystalline Ni_3_Fe and supermalloy powders obtained by mechanical alloying and the commercial nanosized Mn_0.5_Zn_0.5_Fe_2_O_4_ powders were used to produce pseudo core–shell powders that consist of a core formed by very large particles of Ni_3_Fe or supermalloy covered by a discontinuous layer (shell) of nanosized MnZn ferrite.

**Figure 2:**
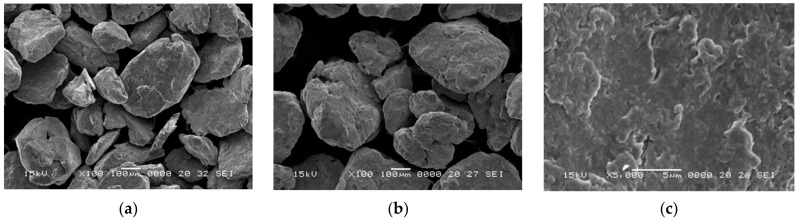

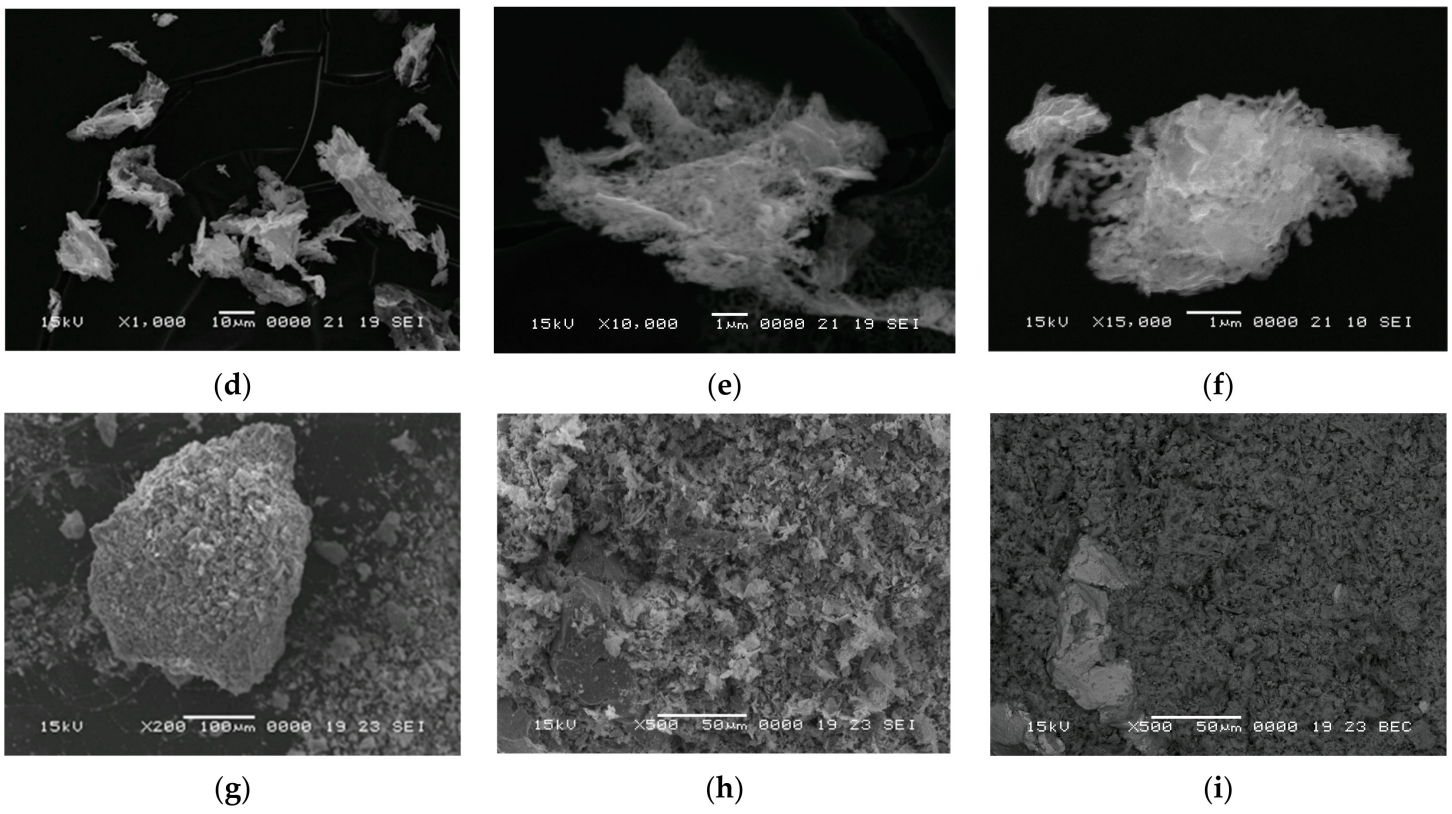
Scanning electron microscopy images of 12 h milled Ni_3_Fe (**a**,**c**), and supermalloy (**b**), nanosized Mn_0.5_Zn_0.5_Fe_2_O_4_ (**d**–**f**) and Ni_3_Fe@Mn_0.5_Zn_0.5_Fe_2_O_4_ pseudo core–shell powders. Images (**a**–**h**) were obtained in secondary electron mode, and (**i**) image was obtained in backscattered electron mode.

The morphology of the powders used for producing Ni_3_Fe@Mn_0.5_Zn_0.5_Fe_2_O_4_ and supermalloy@Mn_0.5_Zn_0.5_Fe_2_O_4_ pseudo core–shell powders, and the Ni_3_Fe@Mn_0.5_Zn_0.5_Fe_2_O_4_ pseudo core–shell powders is presented in Figure 2. The particles of Ni_3_Fe (Figure 2a) and supermalloy (Figure 2b) have similar morphologies. All of the particles have an irregular shape, and the size is from a few tens to a few hundred micrometers. The surface of the particles (see Figure 2c, for N_3_Fe particles) shows that the particles are formed by small particles cold-welded during the milling process. The surface of the metallic particles is rough, which gives a large surface area useful for the physical adherence of nanometric ferrite particles. The Mn_0.5_Zn_0.5_Fe_2_O_4_ powder is presented in Figure 2d–f at magnification up to 15,000×. The particles are agglomerated by flocculation due to their very low size (nanosized particles of 5–100 nm). The pseudo core–shell Ni_3_Fe@Mn_0.5_Zn_0.5_Fe_2_O_4_ are presented in Figure 2g–i. In Figure 2g, it can be seen how a pseudo core–shell particle is formed by a large Ni_3_Fe particle covered by a layer of nanosized MnZn ferrite. Figure 2h,i shows the same particle, but Figure 2h is an image obtained with secondary electrons (SEI), and Figure 2i is an image obtained with backscattered electron contrast (BEC), which put in evidence the different phases from material. Therefore, in both images, it can be observed that, in the bottom left side, there are two small zones uncovered by ferrite particles, which is better shown in the BEC image.

**Figure 3:**
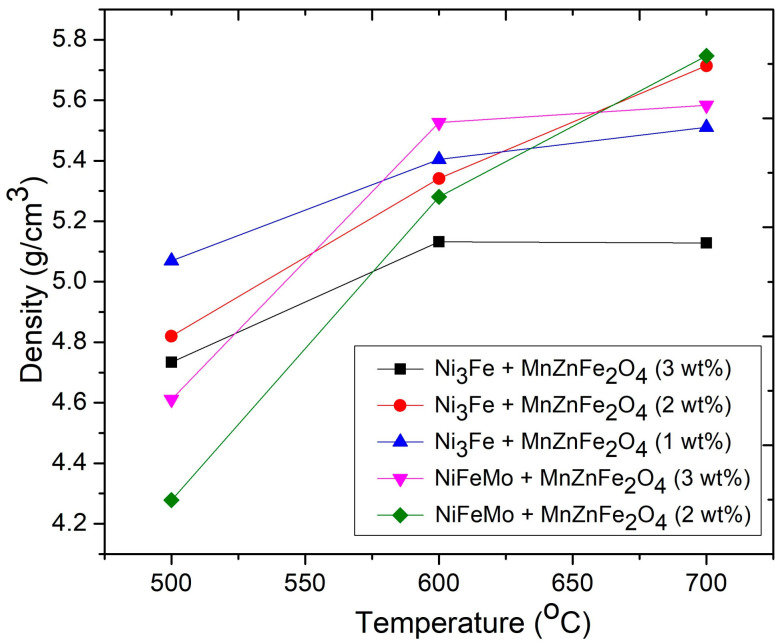
Influence of the amount of ferrite nanoparticles and the sintering temperature on the density of composite compacts obtained by SPS from (N_3_Fe, supermalloy) @Mn_0.5_Zn_0.5_Fe_2_O_4_. Sintering time was 0 min.

**Figure 4:**
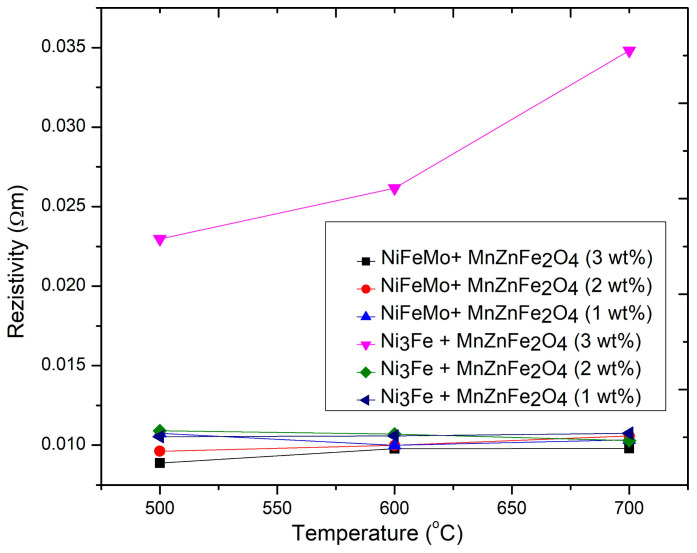
Influence of the amount of ferrite nanoparticles and the sintering temperature on the electrical resistivity of composite compacts obtained by SPS from (N_3_Fe, supermalloy) @Mn_0.5_Zn_0.5_Fe_2_O_4_. Sintering time was 0 min.

The influence of the sintering temperature and the amount of ferrite nanoparticles on the density and electrical resistivity of all composite compacts obtained by SPS from Ni_3_Fe@Mn_0.5_Zn_0.5_Fe_2_O_4_ and supermalloye@Mn_0.5_Zn_0.5_Fe_2_O_4_ pseudo core–shell powders (with 1, 2, and 3 wt.% of ferrite nanoparticles) is shown in Figure 3 and Figure 4, respectively. In Figure 3, it is shown that the composite density increases with increasing sintering time. Concerning the amount of ferrite nanoparticles, for both kind of composites, higher densities were obtained for the composition with 2 wt.% of Mn_0.5_Zn_0.5_Fe_2_O_4_ at sintering temperature of 700 °C. The density of the composite compacts decreases with the increasing of the content of the phase with lower density (ferrite). At lower sintering temperature (500 °C and 600 °C), the density depends strongly on porosity and the pore distribution, which can balance the different densities of the alloy and ferrite phases. This can explain why, at these sintering temperatures, the dependence of the density on ferrite contents for a composite with supermalloy is the opposite of the composite with Ni_3_Fe.

**Figure 5:**
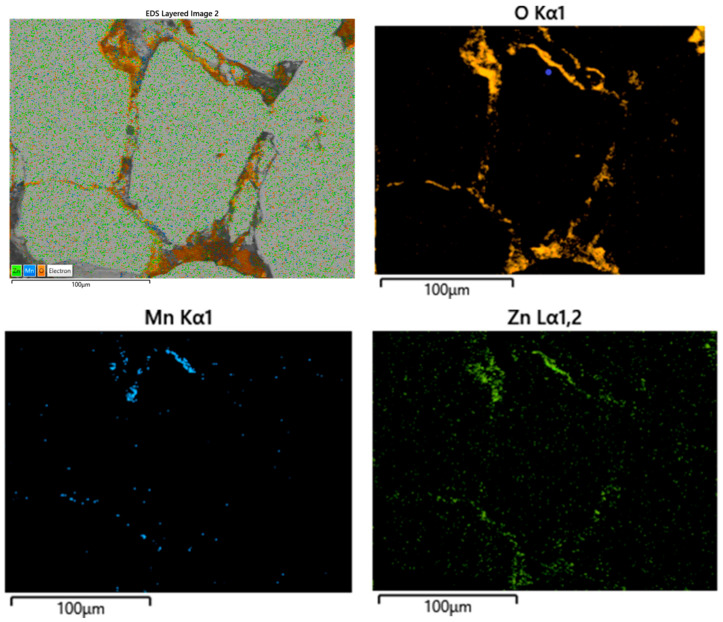
The microstructure and the phase distribution (EDX, elements distribution maps) in the composite compacts obtained from Ni_3_Fe@Mn_0.5_Zn_0.5_Fe_2_O_4_ (3 wt.%) pseudo core–shell powders by SPS at 600 C, 10 min. In the top left side is shown the SEM image of the microstructure with the ferrite elements’ distribution maps superimposed.

The electrical resistivity of the composite SPS-ed compacts is at a magnitude of 1 × 10^−2^ Ωm, 3–4 orders of magnitude higher than supermalloy’s electrical resistivity (4 × 10^−5^ Ωm), due to the presence of a MnZn ferrite layer. The higher electrical resistivity will decline in the same ratio as the eddy currents and the power losses in AC applications. It is important to say that the electrical resistivity depends strongly on the many influencing factors, such as: (i) the continuity of the nano-ferrite network, (ii) the density of the sintered compacts, and (iii) the solid-state reaction between the components during the sintering process, which can produce phases with different electrical resistivities. The electrical resistivity of the composite compacts obtained from the Ni_3_Fe@Mn_0.5_Zn_0.5_Fe_2_O_4_ pseudo core–shell powders with 3 wt.% ferrite nanoparticles is 2–3 times higher than the electrical resistivity of all other composite compacts, at all sintering temperatures. This is in agreement with the lower density of these composite compacts (see Figure 3) and with the better MnZn ferrite network that includes the large particles of ferromagnetic alloy; see Figure 5. Furthermore, the significant change in the electrical resistivity of the samples with 2 wt.% and 3 wt.% compared with the small change in density for the same samples can be explained by the very large difference in the electrical resistivity of permalloy and ferrite (ratio around 1010) compared with the difference in density (ratio around 1.7). By increasing the sintering temperature, the differences in the electrical resistivity of all compacts (excepting Ni_3_Fe@Mn_0.5_Zn_0.5_Fe_2_O_4_, 3 wt%) are diminished, and the electrical resistivity is around 1 × 10^−2^ Ωm for all compacts. This can be due to the changes in the phase composition of the sintered compacts by a solid-state reaction during sintering, changes that homogenize the phase composition of the compacts, as will be shown later by the XRD patterns of the sintered compacts.

The microstructure and the phases (alloy and ferrite) distribution in the structure of the SPS-ed composite compacts were determined by scanning electron microscopy and X-ray microanalysis (EDX). In order to obtain the very low power losses in AC applications, the microstructure of the composite compacts should consists of large ferromagnetic particles embedded in a continuously dielectric network, in our case a ferrite network. The ferrite network assures the high electrical resistivity, and the large ferromagnetic particles assure the high magnetic properties. The microstructure and the phase distribution, alloy and MnZn ferrite, in the composite compacts obtained from Ni_3_Fe@Mn_0.5_Zn_0.5_Fe_2_O_4_ (3 wt.% nano ferrite) pseudo core-shell powders by SPS at 600 °C, 10 min is shown in Figure 5. In the top left side is shown an SEM image of the composite microstructure with the elements’ distribution maps superimposed. The phase distribution in the microstructure is evidenced by the chemical elements’ distribution maps (determined by EDX). In the figure are shown only the distribution maps of O, Mn, and Zn, the elements that indicate the presence of MnZn ferrite. It can be seen in Figure 5 that the MnZn ferrite embeds large particles of Ni_3_Fe (permalloy). Furthermore, some pores are shown in Figure 5, the gray zones in the microstructure uncovered by the element’s distribution maps.

**Figure 6:**
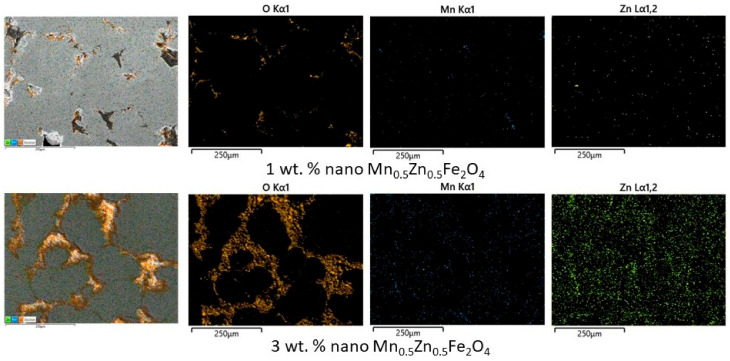
The influence of amount of nano MnZn ferrite on the microstructure of the Ni_3_Fe/Mn_0.5_Zn_0.5_Fe_2_O_4_ SPS-ed composite compacts. Sintering parameters: sintering temperature—700 °C, sintering time—0 min. On the left side is the SEM image over which the O, Mn, and Zn distribution maps are superimposed.

The microstructure and the phase distribution in the composite compacts with different amounts of MnZn ferrite (1 wt. % and 3 wt. %) are shown in Figure 6. The O, Mn, and Zn distribution maps are superimposed on the SEM image of the microstructure (left side). For the composite with 1 wt. % of nanosized MnZn ferrite, it can be seen that there are many large particles of Ni3Fe in contact. Oppositely, for the composite cu 3 wt. % of nanosized MnZn ferrite, it can be seen that the ferrite network is too thick. This can have a negative influence on the composite magnetic properties by reducing the ferromagnetic alloy amount, by reducing compact density, and by increasing the compaction difficulty. Furthermore, the microstructure of the sintered compact with 1 wt. % of MnZn ferrite contains more pores that the other compositions.

**Figure 7:**
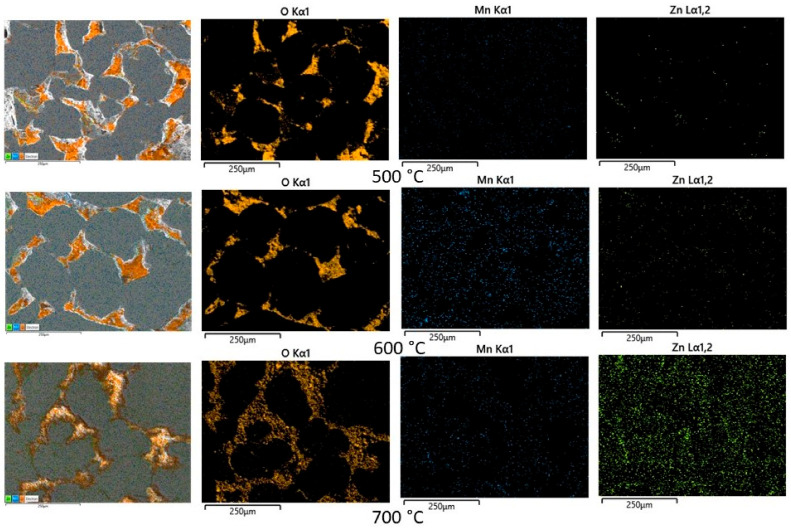
The influence of the sintering temperature on the microstructure of the Ni_3_Fe/Mn_0.5_Zn_0.5_Fe_2_O_4_ (3 wt. %) SPS-ed composite compacts. Sintering time—0 min. On the left side of each series is the SEM image over which the O, Mn, and Zn distribution maps are superimposed.

The influence of the sintering temperature on the microstructure and on the phase distribution is presented in Figure 7, for the case of the composite compacts Ni3Fe/Mn0.5Zn0.5Fe_2_O_4_ with 3 wt. % of MnZn ferrite. For all the sintering temperatures, the phase distribution, as is shown by the O, Mn, and Zn distribution maps, seems to be similar. For the composites SPS-ed at temperatures of 500 and 600 °C, the microstructure contains some pores, more for the sintering temperature of 500 °C. The microstructure of the composite compacts sintered at 700 °C seems to not have pores. However, the ferrite network seems to be damaged by the loss of some oxygen atoms that could migrate to the alloy particles through a solid-state reaction, as will be shown by XRD patterns (see Figure 10 and Figure 11).

**Figure 8:**
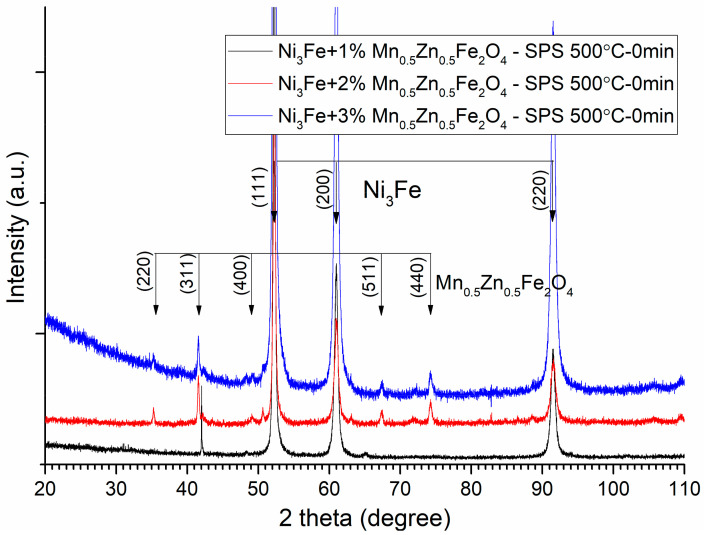
XRD patterns of the Ni_3_Fe/Mn_0.5_Zn_0.5_Fe_2_O_4_ composite compacts with 1, 2, and 3 wt.% of MnZn ferrite obtained by SPS at sintering temperature of 500 °C. For clarity, the XRD patterns have been shifted vertically.

**Figure 9:**
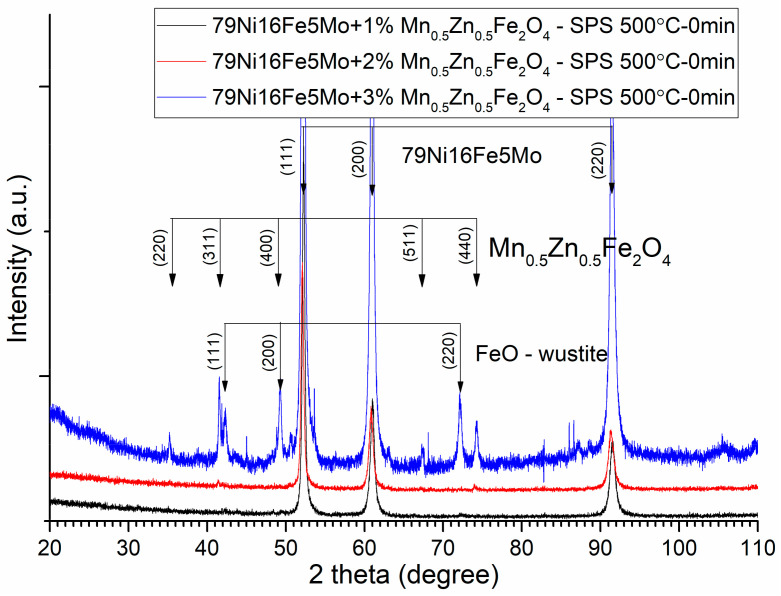
XRD patterns of the supermalloy/Mn_0.5_Zn_0.5_Fe_2_O_4_ composite compacts with 1, 2, and 3 wt.% of MnZn ferrite obtained by SPS at sintering temperature of 500 °C. For clarity, the XRD patterns have been shifted vertically.

**Figure 10:**
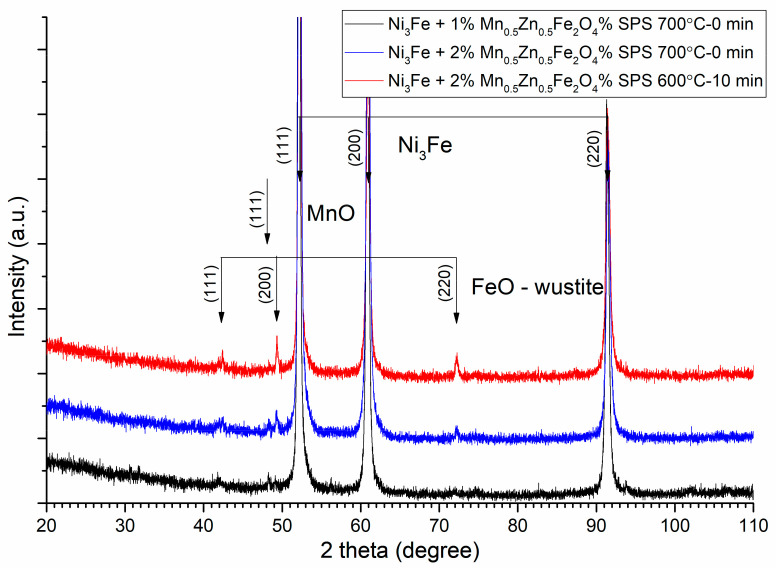
XRD patterns of the Ni3Fe/Mn0.5Zn0.5Fe2O4 composite compacts with 1 and 2 wt.% of MnZn ferrite obtained by SPS at sintering temperatures of 600 and 700 °C and sintering time of 0 min and 10 min. For clarity, the XRD patterns have been shifted vertically.

**Figure 11:**
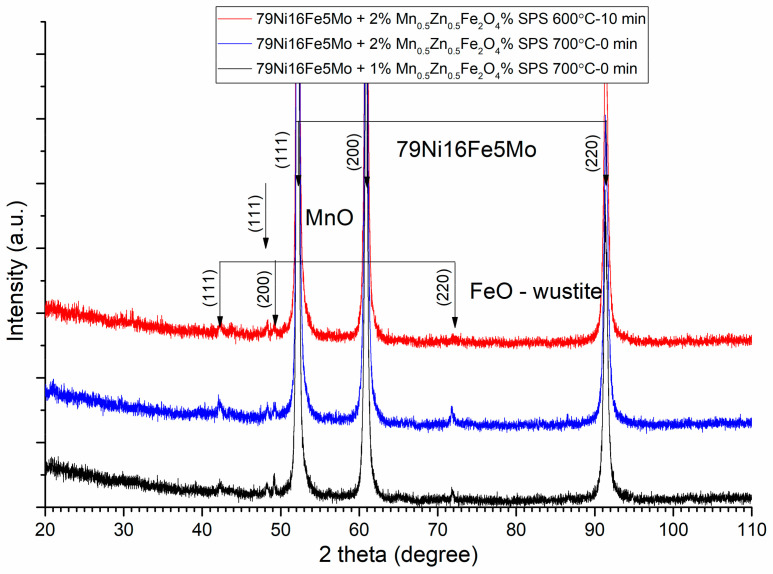
XRD patterns of the supermalloy/Mn_0.5_Zn_0.5_Fe_2_O_4_ composite compacts with 1 and 2 wt.% of MnZn ferrite obtained by SPS at sintering temperatures of 600 and 700 °C and sintering time of 0 min and 10 min. For clarity, the XRD patterns have been shifted vertically.

The sintering parameters, time, and temperature must be chosen correctly in order to avoid the solid-state reaction between the initial phases (alloy and ferrite) of the composite compacts. This can be checked through the X-ray diffraction of the sintered compacts. The XRD patterns of the Ni_3_Fe/Mn_0.5_Zn_0.5_Fe_2_O_4_ composite compacts and supermalloy/Mn_0.5_Zn_0.5_Fe_2_O_4_ composite compacts with 1, 2, and 3 wt.% of MnZn ferrite obtained by SPS at sintering temperature of 500 °C, 0 min holding time, are presented in Figure 8 and Figure 9, respectively. From both Figure 8 and Figure 9, it can be remarked that, at these sintering conditions, the initial phases, alloy and ferrite, are conserved in the microstructure of the sintered compacts. By increasing the sintering temperature or/and the sintering time, a solid-state reaction occurs between the initial phases that compose the pseudo core–shell powders. This can be observed in Figure 10 and Figure 11, where the Bragg maxima of the MnZn_0.5_Fe_2_O_4_ spinel phase vanishes and the Bragg maxima of the other phases are present, due to the solid-state reaction during sintering. Therefore, in Figure 10 and Figure 11, it can be observed the Bragg maxima of the FeO, and MnO. The FeO and MnO phases are a consequence of the solid-state reaction between Ni–Fe alloy and MnZn ferrite. The Fe is more reactive with the oxygen compared to Zn, and therefore, the Fe atoms from the alloys react with ferrite and decomposed it at higher sintering temperatures [14,16]. All these new undesired phases occurred during sintering negatively affect the magnetic properties (permeability and magnetization) of the sintered composite compacts. Therefore, it is important to realize a compromise between the necessity to conserve the initial phases of the composite (low sintering time and sintering temperature) and the goal to have high density, high electrical resistivity, high permeability up to high frequencies, low power losses in AC applications, and high magnetization, which can be obtained by sintering at high temperature and a long sintering time.

**Figure 12:**
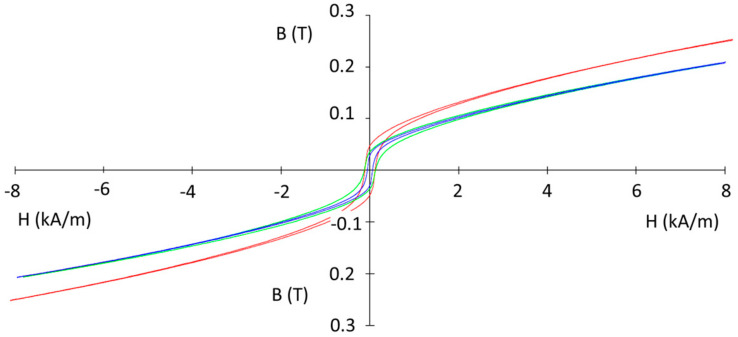
The B–H hysteresis curves in DC of the (Ni_3_Fe, supermalloy)/Mn_0.5_Zn_0.5_Fe_2_O_4_ composite compacts with 1 and 2 wt. % of MnZn ferrite, SPS-ed at temperature of 500 °C, 0 min holding time.

**Figure 13:**
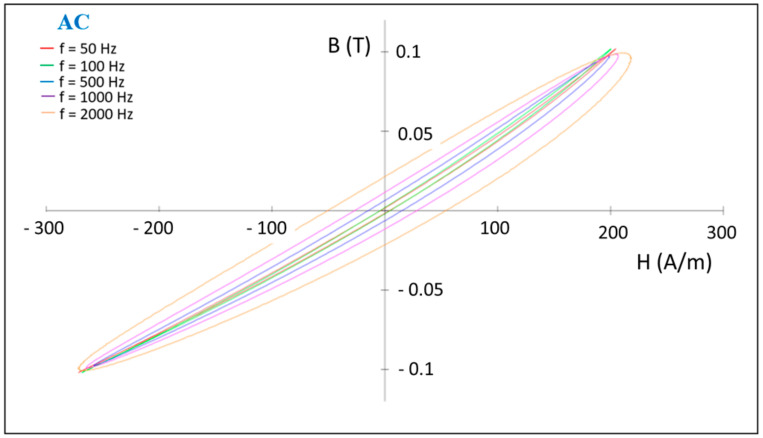
The influence of the frequency on the B–H hysteresis curves of the supermalloy/Mn**_0.5_**Zn**_0.5_**Fe_2_O_4_ composite compacts with 1 wt. % of MnZn ferrite, SPS-ed at temperature of 500 °C, 0 min holding time. The induction level was seated at 0.01 T.

**Figure 14:**
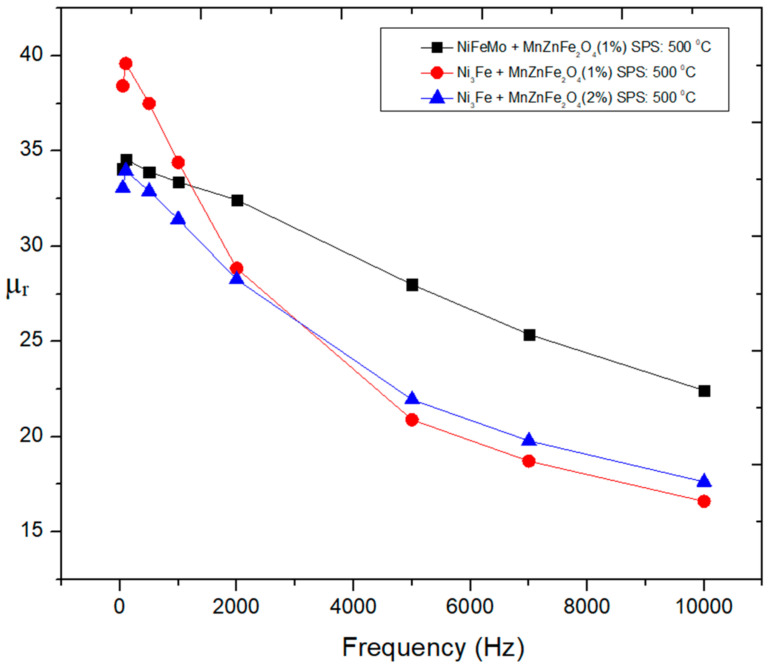
Evolution of initial magnetic permeability as a function of the frequency for toroidal-shaped (Ni_3_Fe, supermalloy)/Mn**_0.5_**Zn**_0.5_**Fe_2_O_4_ composite compacts, SPS-ed at the sintering temperature of 500 °C. The induction level was set at 0.01 T.

**Figure 15:**
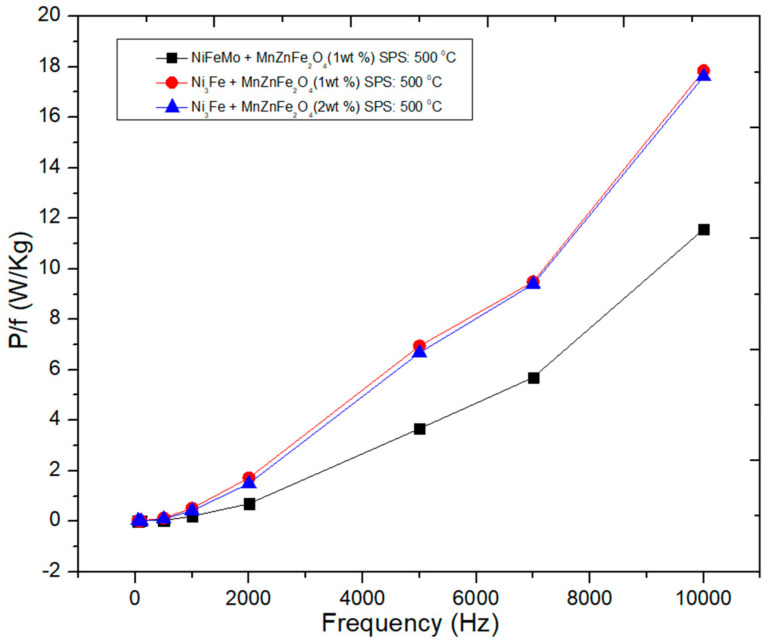
Evolution of the power losses as a function of the frequency for toroidal-shaped (Ni_3_Fe, supermalloy)/Mn**_0.5_**Zn**_0.5_**Fe_2_O_4_ composite compacts, SPS-ed at the sintering temperature of 500 °C. The induction level was set at 0.01 T.

The magnetic properties of the sintered composite compacts produced from the Ni_3_Fe@Mn_0.5_Zn_0.5_Fe_2_O_4_ and supermalloy@Mn_0.5_Zn_0.5_Fe_2_O_4_ pseudo core–shell powders were determined in DC and in AC up to 10,000 Hz. The hysteresis curves in DC, in Figure 12, for some (Ni_3_Fe, supermalloy)/Mn_0.5_Zn_0.5_Fe_2_O_4_ composite compacts with 1 and 2 wt. % of MnZn ferrite, SPS-ed at low temperature for conserving the initial phases of the composite, give information about the coercive field, remanence, and saturation induction. The coercive field is in the range of 58 to 126 A/m, the lower coercive field being for the supermalloy)/Mn**_0.5_**Zn**_0.5_**Fe_2_O_4_ composite with 1 wt.% of MnZn ferrite. The remanence induction is low, and the saturation tendence of the composite is also low. The best results concerning the remanence and saturation were obtained for the Ni_3_Fe/Mn**_0.5_**Zn**_0.5_**Fe_2_O_4_ with 1 wt. % of MnZn ferrite. The behavior in AC measured for B = 0.01 T at a frequency up to 2000 Hz is shown in Figure 13 for the supermalloy/MnZnFe_2_O_4_ composite compacts with 1 wt. % of MnZn ferrite, SPS-ed at low temperature (500 °C) and 0 holding time. Again, the results are not as expacted, due to the low density, high porosity, and low electrical resistivity of the compacts sintered in these conditions; see Figure 3, Figure 4 and Figure 7. For example, initial permeability is low (around 35), and it remain constant up to 2000 Hz. As known, the permeability strongly depends on the material density, porosity, and non-magnetic phases [10,21,22]. The evolution of the initial relative permeability and of the power losses versus frequency is shown in Figure 14 and Figure 15, respectively. The best relative initial permeability, around 40, was obtained for the Ni_3_Fe/Mn**_0.5_**Zn**_0.5_**Fe_2_O_4_ composite with 1 wt. % of MnZn ferrite. The relative initial permeability decreases linearly for frequencies larger than 2000 Hz.

Concerning the level and the evolution of the power losses versus frequency the results are promising; see Figure 15. Up to a frequency of 2000 Hz, the power losses are lower than 2 W/kg for an induction level of 0.01 T. Up to 10 kHz, the power losses increase exponentially, but they remain at a low level, lower than 18 W/Kg, even lower than 12 W/kg for the supermalloy)/Mn_0.5_Zn**_0.5_**Fe_2_O_4_ composite with 1 wt.% of MnZn ferrite sintered composite.

## 4. Conclusions

The pseudo core–shell powders like (Ni-Fe alloy)@Mn_0.5_Zn_0.5_Fe_2_O_4_ consisting of a core of large ferromagnetic particles surrounded by a discontinuous layer of nanosized MnZn ferrite were successfully obtained. The SPS-ed composite compacts obtained from these pseudo core–shell powders show the electrical and magnetic properties in DC and AC magnetic fields that strongly depend on the compact’s density, on the quality of the ferrite network in the microstructure, and on the possibility of avoiding a solid-state reaction between the alloy and ferrite phases during the sintering process. Sintering at 500 °C with 0 min holding time conserves the initial phases of the composite, but the density of the compacts is too low to assure the good magnetic properties. Oppositely, a sintering at 700 °C for 0 min holding time or at 600 °C for 10 min holding time can not avoid a solid-state reaction between the alloy and ferrite, and some undesired phases like FeO and MnO appears to have a damaged microstructure and diminished magnetic properties. Therefore, it was proven that it is important to find a compromise to conserve the initial phases in the composite by sintering at low temperature for a short sintering time and to obtain a high density by sintering at a higher temperature for a longer holding time. The amount of the nanosized MnZn ferrite should be lower than 2 wt. % to obtain a quasi-continuously ferrite network with a sufficient thickness to obtain high electrical resistivity. The electrical resistivity of the composite compacts is 3–4 order of magnitude higher than supermalloy electrical resistivity, and it strongly depends not only on the amount of MnZn ferrite but also on the microstructure of the composite, especially on the continuity of the nano-ferrite network. The influence of the sintering parameters on the magnetic properties is not relevant now, due to the appearance of new phases during sintering. However, it can be seen that the best initial permeability, around 40, was obtained for the Ni_3_Fe/Mn_0.5_Zn_0.5_Fe_2_O_4_ composite with 1 wt. % of MnZn ferrite, sintered at a temperature of 500 °C with a holding time of 0 min. Furthermore, for the same sintering conditions, the power losses were lower than 2 W/kg up to 2000 Hz, for B = 0.01 T.

## Data Availability

The authors confirm that the data supporting the findings of this study are available within the article.
